# A novel strategy for stabilization of sub-nanometric Pd colloids on kryptofix functionalized MCM-41: nanoengineered material for Stille coupling transformation

**DOI:** 10.1038/s41598-021-97914-z

**Published:** 2021-09-16

**Authors:** Hassan Alamgholiloo, Nader Noroozi Pesyan, Sadegh Rostamnia

**Affiliations:** 1 Department of Organic Chemistry, Faculty of Chemistry, Urmia University, 57159 Urmia, Iran; 2 Organic and Nano Group (ONG), Department of Chemistry, Iran University of Science and Technology (IUST), PO Box, 16846-13114 Tehran, Iran

**Keywords:** Chemistry, Materials science

## Abstract

The stabilization of sub-nanometric metal particles (< 1 nm) with suitable distribution remained challenging in the catalytic arena. Herein, an intelligent strategy was described to anchoring and stabilizing sub-nanometric Pd colloids with an average size of 0.88 nm onto Kryptofix 23 functionalized MCM-41. Then, the catalytic activity of Pd@Kryf/MCM-41 was developed in Stille coupling reaction with a turnover frequency (TOF) value of 247 h^−1^. The findings demonstrate that porous MCM-41 structure and high-affinity Kryptofix 23 ligand toward adsorption of Pd colloids has a vital role in stabilizing the sub-nanometric particles and subsequent catalytic activity. Overall, these results suggest that Pd@Kryf/MCM-41 is a greener, more suitable option for large-scale applications and provides new insights into the stabilization of sub-nanometric metal particles.

## Introduction

Within the field of silica-based materials, MCM-41 as a nanoreactor has possessed much attention in the catalytic process arena due to unique properties such as high surface areas, chemical stability, and structural rigidity^1–3^. Furthermore, these porous materials can be an excellent candidate for the stabilization of metal nanoparticles (NPs) as a heterogeneous catalyst in modern synthesis^4–6^. Recently, efforts have been made for the fabrication of metal NPs in MCM-41, and these nanostructures demonstrated potential utility in catalytic processes^7–9^. Therefore, a simple, inexpensive, and efficient synthetic method that facilitates the immobilization of metal NPs in MCM-type solid materials is required.

The cryptand compounds known also with the commercial name Kryptofix are frequently used in host–guest chemistry^10–14^. 1,4,7,13,16-Pentaoxa-10,19-diazacycloheneicosane (Kryptofix 23) as a specific class of aza-crown ether have been known for their high affinity to bind spherical cations. They are macro-bicyclic ligands that contain an internal cavity of about spherical shape and provide very stable and selective complexes with a variety of metal cations^15^. These ligands are exceptionally versatile in selectively binding a range of metal ions and led to the development of host–guest chemistry.

Recently, the application of colloidal metal nanoparticles has become a promising approach for catalytic applications. Various techniques such as capping agents, surfactants, and organic ligands have been applied for the preparation and stability of colloidal nanoparticles to overcome aggregate problems^16–19^. Among the methods mentioned above, crown ether macrocyclic compounds have an excellent capacity to adsorb metal ions^20–23^. Therefore, it is an excellent candidate for the anchoring and stabilization of colloidal metal nanoparticles. Recently, Verport and co-workers developed the loading of metallic Pd nanocrystals on azobenzene-based colloidal porous organic polymer for photocatalytic Suzuki coupling reaction^24^. In parallel, Shi and co-workers explored the synthesis of Pd colloids grafted mesoporous silica materials SBA-15 in Mizoroki–Heck reactions^25^. Moreover, the authors’ previous study^17,19,26–28^ and other studies^29–37^ have proven that colloidal Pd, Pd NPs, and Pd complex can greatly boost C–C bond formations.

Considering the unique metal colloid structure, we have envisioned an intelligent method for anchoring and stabilizing sub-nanometric Pd onto Kryptofix 23 functionalized MCM-41. We also demonstrated the catalytic activity of this nanostructure in the carbon–carbon coupling reaction. To the best of our knowledge, this is the first example of the stabilization of sub-nanometric Pd colloids with crown ether macrocyclic compounds that exhibit enhanced catalytic activity in the Stille coupling reaction. The current study widens the applications of sub-nanometric metal particles for catalytic process.

## Experimental

### Synthesis of Kryf/MCM-41 mesoporous

The nanoporous MCM-41 was prepared with a sol–gel method according to our previous report^38,39^ (Detailed in Support Information). Initially, 2.0 g calcinated MCM-41 was dissolved in 30 mL toluene solutions under the reflux condition at 12 h. Then, 10 mmol (2.4 mL) (3-chloropropyl) triethoxysilane was added to the mixture reaction under stir for 24 h. The prepared powder from the reaction was washed with ethanol several times, which obtain MCM-41-Cl with loading 56.5% linker. After that, 0.5 mL of Kryptofix 23 and 1.0 ml Et_3_N in 20 ml EtOH was added to 1.5 g MCM-41-Cl under reflux condition. After stirring for 24 h, the mixture was washed several times with EtOH. Finally, it was dried in a vacuum at 80 °C for 12 h, and a 61% yield of Kryf/MCM-41 was synthesized.

### Preparation of Pd@Kryf/MCM-41

The colloidal Pd NPs was synthesized by using the impregnation reduction method according to our previous report with some modification^19^. First, 0.02 g Pd (OAc)_2_ (Pd: 47.5%) and 0.2 g polyvinyl alcohol PVA was dispersed in 15 mL MeOH under sonication for 30 min. Subsequently, 5.0 mL 0.1 M of NaBH_4_ was poured into the mixture mentioned above to fabricate a colloidal Pd solution. After stirring for 60 min under ambient conditions, 1.0 g Kryf/MCM-41 was added to the mixture. After stirring for 4 h at ambient conditions, the final solid result was collected by filtration and washed with EtOH several times then dried at room temperature. Wt% of Pd in the Kryf/MCM-41 mesoporous was calculated via inductively coupled plasma optical emission spectrometry (ICP-OES), and the resultant yield indicated 0.72%.

### General procedure for Stille coupling reactions

In the model reaction, the mixture of aryl halides (3 mmol), triphenyltin chloride (1 mmol), Na_2_CO_3_ (3 mmol), polyethylene glycol-400 (PEG-400, 2 mL) was added to 0.36 mol% Pd@Kryf/MCM-41 catalyst. The reaction vessel was capped and allowed to stir at room temperature for 3 h. After the reaction is complete, the organic phase was extracted with ethyl acetate/petroleum ether (1:2) and dried over sodium sulfate (Na_2_SO_4_) to obtain the corresponding biphenyl. Finally, all biaryls were identified by ^1^H and ^13^C-NMR spectroscopy and comparison with the literature^28,40^.

## Results and discussion

**Figure 1:**
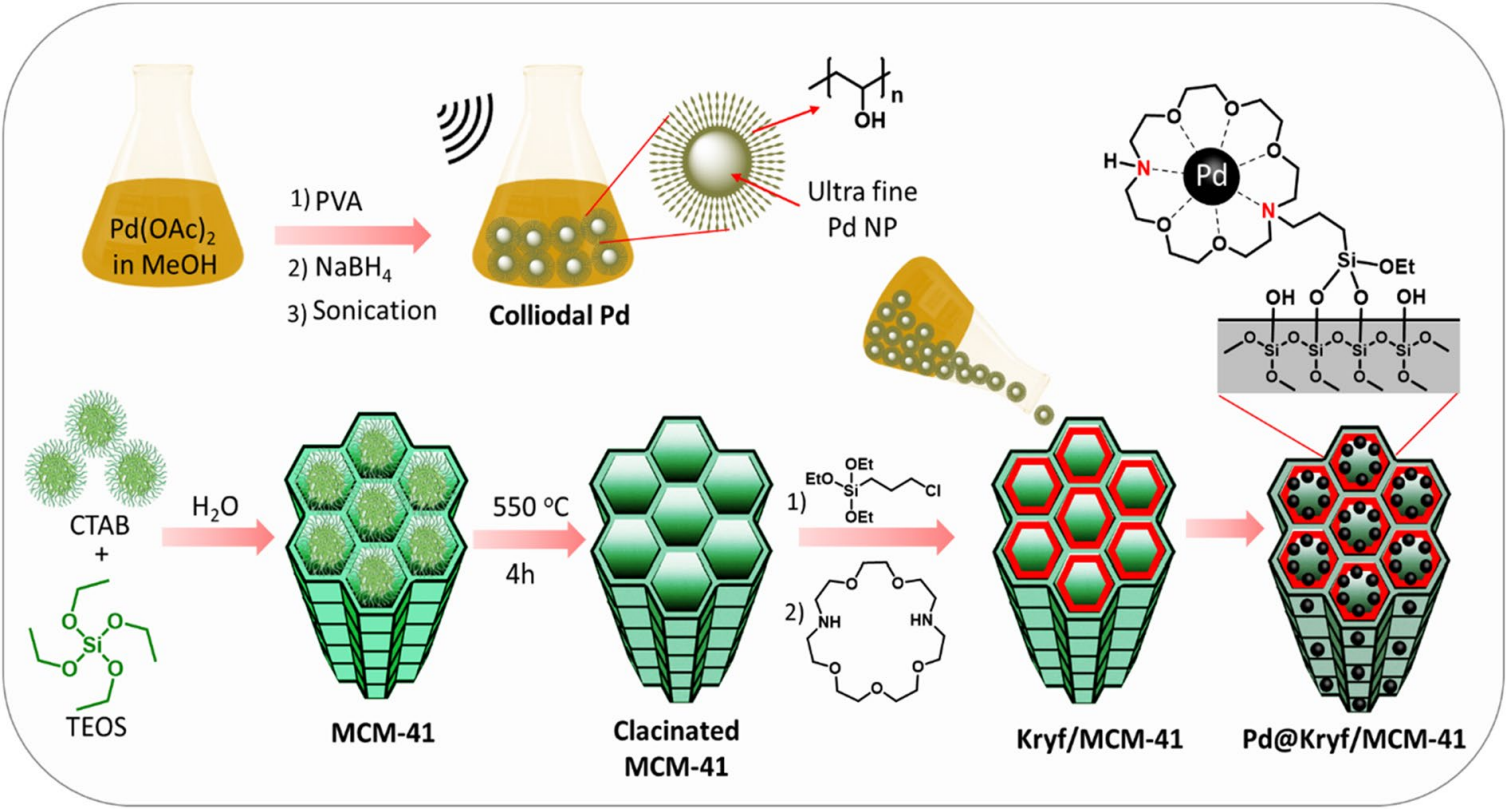
Synthetic pathway of Pd@Kryf/MCM-41.

In addition to the scientific findings in introducing engineered nanomaterials^41–46^, herein, Pd@Kryf/MCM-41 mesoporous silica was presented for Stille coupling reaction (Fig. 1). Our initial efforts were geared towards preparing MCM-41 mesoporous silica material via the sol–gel method, which was followed by grafting Kryptofix 23 into pores of MCM-41. Finally, Kryf/MCM-41 was then utilized as excellent support for immobilizing colloidal Pd NPs in the sub-nanometer scale.

### Structural and morphology characterization

**Figure 2:**
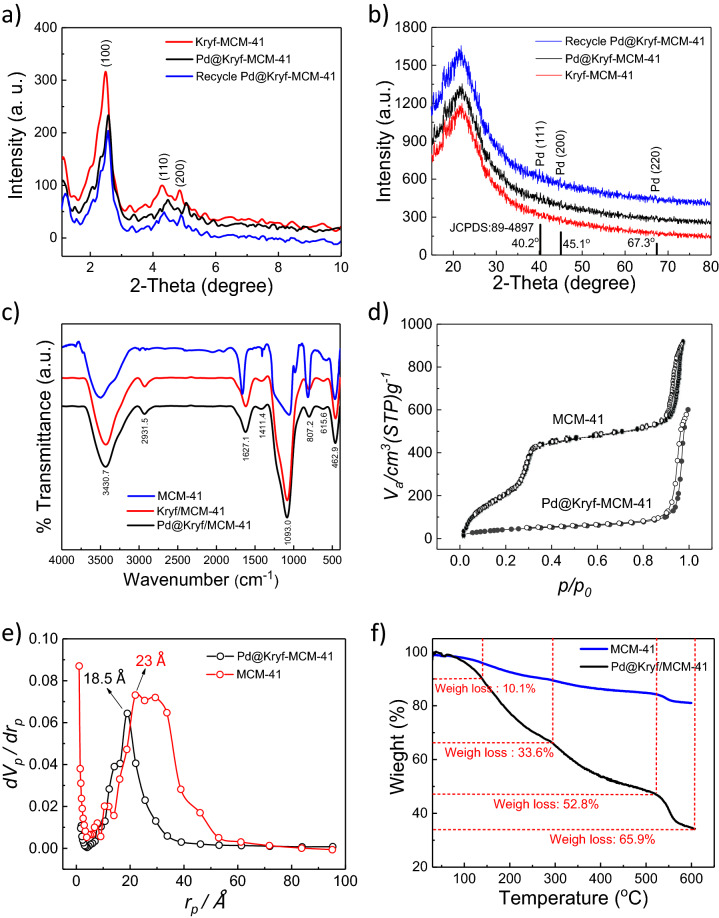
SA-XRD and WA-XRD patterns for Kryf/MCM-41, Pd@Kryf/MCM-41 and recycled Pd@Kryf/MCM-41 (**a**, **b**); FT-IR of MCM-41, Kryf/MCM-41, and Pd@Kryf/MCM-41 (**c**); N_2_ adsorption–desorption profile (**d**); BJH plot (**e**); and TGA curves of MCM-41 and Pd@Kryf/MCM-41 (**f**).

First, the crystalline structure of Kryf/MCM-41 and Pd@Kryf/MCM-41 was analyzed using the powder XRD pattern. In a small-angle XRD (SA-XRD) pattern of Kryf-MCM-41, the characteristic diffraction peaks at 2.3–4.7° for the planes (100), (110), and (200) can be indexed 2D-hexagonal p6mm pore MCM-41 structure^47^, while these peaks in Pd@Kryf/MCM-41 was reduced and slightly shifted to relatively high 2θ region^5,48^ (Fig. 2a). In a wide-angle XRD (WA-XRD) pattern, a broad peak that appeared under the condition 2θ = 23.9° is consistent with the amorphous nature of the pore wall of MCM-41 structure^49,50^. Also, three peaks that appeared under the condition 2θ = 40.2°, 45.1°, and 67.3° are consistent with those of the crystal planes of Pd NPs (JCPDS 89–4897) (Fig. 2b). Furthermore, according to the Scherrer equation, the average crystalline size of Pd was found to be ~ 0.88 nm (Table S1, ESI), which demonstrated that sub-nanometric particles. Moreover, the formation of sub-nanometric Pd was confirmed further by and high-resolution TEM (HR-TEM) and STEM mapping microscopy.

Subsequently, chemical groups of these meso-structures were studied by Fourier transform infrared (FT-IR) spectra (Fig. 2c). The peaks observed at 3430 cm^−1^ and 1627 cm^−1^ can be attributed to hydroxyl groups and adsorbed water^51^, respectively. The absorption band in 2931 cm^−1^ can be ascribed to the CH_2_-stretching vibration peak of Kryptofix 23 and the *n*‐propyl aliphatic chain. Also, the peaks observed at 1411 cm^−1^ after the functionalization of MCM-41 with Kryptofix 23 confirms the covalent bonding crown ether ligand to the surface mesoporous silica.

The porosity properties of Pd@Kryf/MCM-41 were measured using the isotherm and Brunauer–Emmett–Teller (BET) plot. MCM-41 had a surface area of 990 cm^2^/g^−1^, a pore volume of 1.306 cm^3^/g, and pore size of 4.6 nm (Table S2, ESI). Meanwhile, Pd@Kryf/MCM-41 surface area, pore volume, and pore size were 601 cm^2^/g^−1^, 0.886 cm^3^/g, and 3.7 nm, which reflects the presence of the meso-size pores (Fig. 2d,e). Moreover, decrease surface area Pd@Kryf/MCM-41 was attributed to the loading of crown ether compound and Pd NPs.

TGA curves of MCM-41 and Pd@Kryf/MCM-41 demonstrate the weight loss of the organic material and thermal stability of Kryptofix 23 ligand in MCM-41. Figure 2f illustrates four weight loss steps in the TGA curve of Pd@Kryf/MCM-41 catalysts. The first weight loss (10.1%) between 30 and 140 °C is occurred due to the removal of adsorbed water moisture at the surface. The next weight loss (33.6%) from 140 to 295 °C is due to the decomposition of organic material and burning of ligand, which can be found that Kryptofix 23 material is relatively stable below 295 °C. After 295 °C, the phase remains completely changed, which was stable up to over 510 °C.

**Figure 3:**
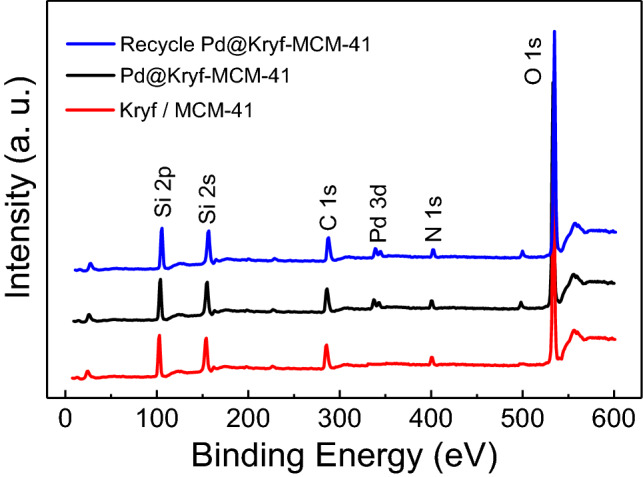
XPS spectra of Kryf/MCM-41, Pd@Kryf/MCM-41, and recycled Pd@Kryf/MCM-41.

The interaction between Pd NPs with Kryf/MCM-41 was further investigated by XPS spectrum (Fig. 3). As for Kryf/MCM-41 meso-structure, the C 1s and N 1S XPS spectra in 285.5 and 399.1 eV, indicating to the thoroughly immobilized Kryptofix 23 ligand in MCM-41 surface. Meanwhile, the appearance of the Pd 3d XPS spectrum in 336–342 eV, confirming the successful coordination of colloidal Pd NPs in Kryf/MCM-41.

**Figure 4:**
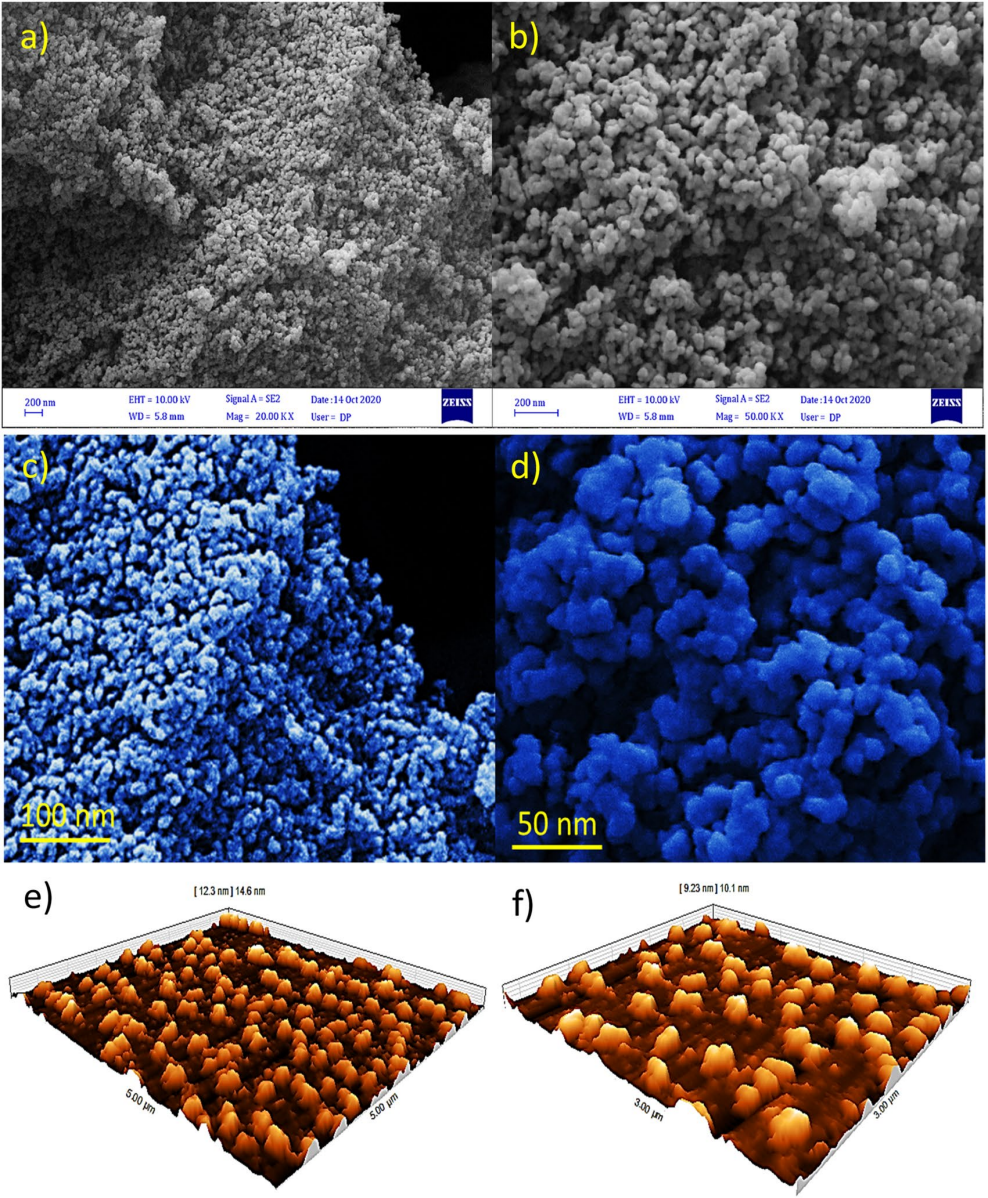
FESEM micrograph (**a**, **b**); colored FESEM (**c**, **d**); AFM images of Kryf/MCM-41 and Pd@Kryf/MCM-4 1 (**e**, **f**).

Field emission scanning electron microscopy (FESEM) image of the prepared Kryf/MCM-41 and Pd@Kryf/MCM-41 shows round-shaped particles with a mean diameter of ~ 22 nm (Fig. 4a,b). After the deposition of colloidal Pd NPs on the surface of Kryf/MCM-41, the structures of the round-shaped particles were preserved, indicating that NPs were anchored in an orderly manner (Fig. 4b). The surface of MCM-41 with Kryptofix 23 ligand helped stabilize Pd NPs and inhibition the agglomeration. Besides, the round-shaped morphology of Kryf/MCM-41 and Pd@Kryf/MCM-41 was further confirmed with colored FESEM and Atomic Force Microscope (AFM) (Fig. 4c–f).

**Figure 5:**
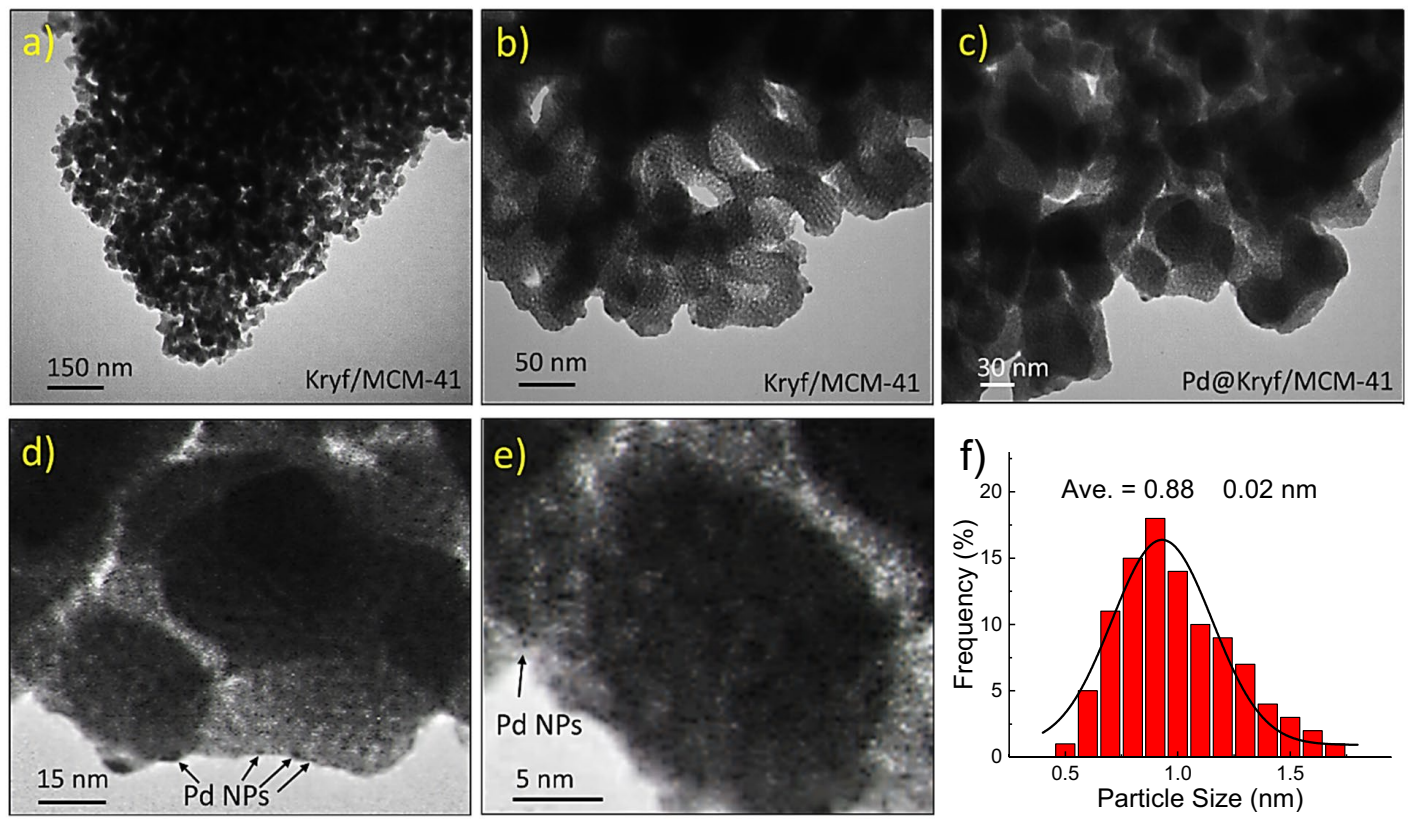
TEM images of Kryf/MCM-41 (**a**, **b**); Pd@Kryf/MCM-41 (**c**, **d**); HR-TEM of Pd@Kryf/MCM-41 (**e**); and corresponding size distributions of Pd NPs (**f**).

The Pd particles are not observable using FESEM measurements, so transmission electron microscopy (TEM) was employed to elucidate the morphology further and loading NPs on the mesoporous silica surface. As depicted in the TEM images, Kryf/MCM-41 and Pd@Kryf/MCM-41 possessed distinct regular channeling pores of 2D-hexagonal pore structure (Fig. 5a–c), which is confirmed with XRD pattern. The high magnifier image (~ 15 nm, Fig. 5d) indicates the formation of NPs in Kryf/MCM-41 surface without any agglomeration. When only MCM-41 was used for the deposition instead of Kryptofix 23 functionalized, an agglomeration of Pd NPs with relatively bigger particle sizes were observed on the surface of MCM-41 (Fig. S1, ESI). A close inspection of HR-TEM image confirms the formation of NPs (Fig. 5e). Moreover, the statistical count of the particle size of Pd (> 100 counts and Gauss fit) in Kryf/MCM-41 surface was determined to be 0.88 ± 0.02 nm (Fig. 5f).

**Figure 6:**
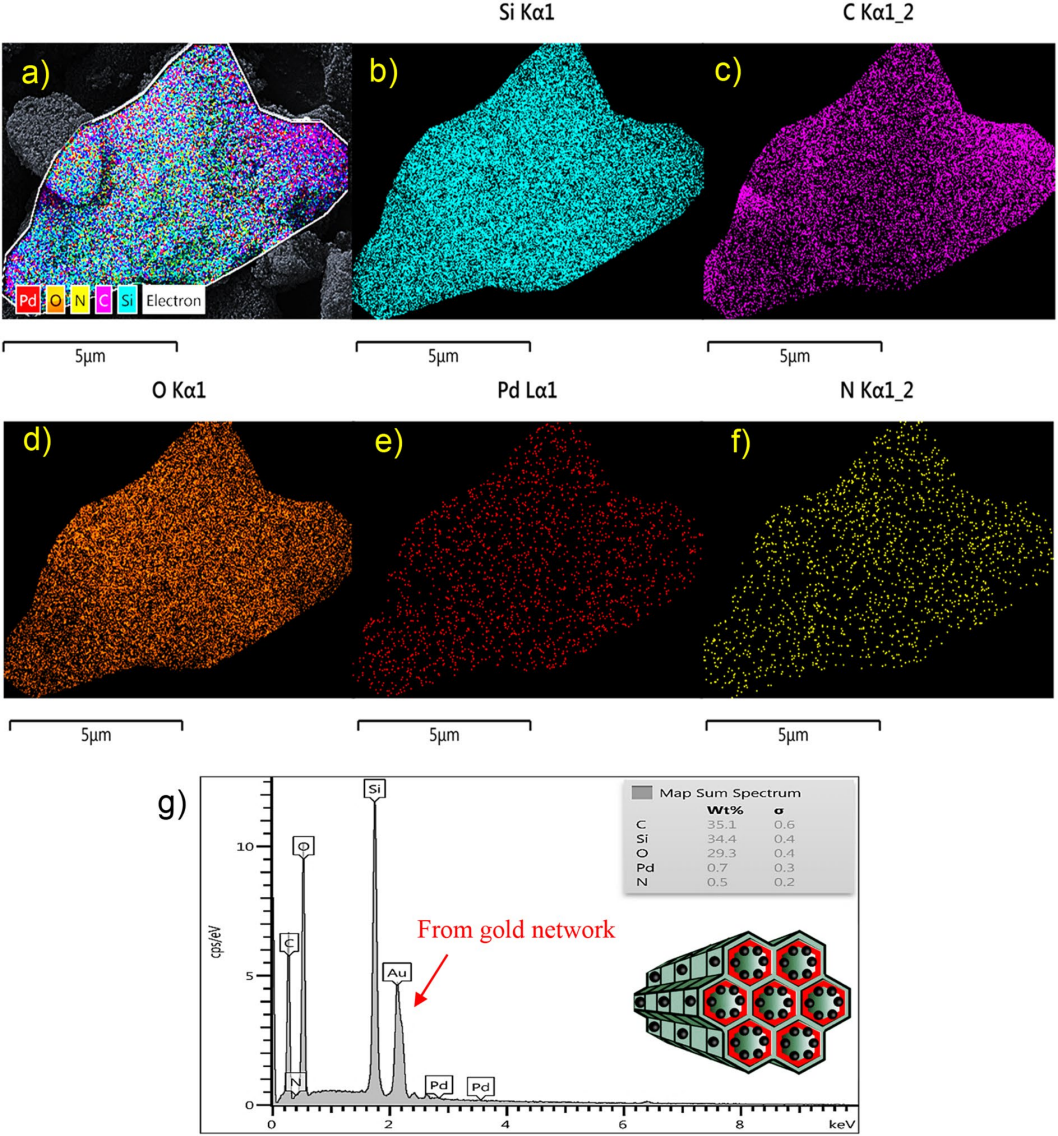
STEM/EDS-mapping micrograph (**a**–**f**) and EDX analysis of Pd@Kryf/MCM-41 (**g**).

In addition to FESEM and TEM images, the STEM spectrum exhibit more detail about the deposition of Pd NPs onto the mesoporous material (Fig. 6a–f). The dark-field STEM images of Pd@Kryf/MCM-41 can be affirmed that the Pd particles are homogeneously deposited onto the Kryf/MCM-41 mesoporous. Meanwhile, STEM mapping images and energy dispersive X-ray (EDX) analysis, confirm the presence of all constituent elements (i.e., Si, O, C, N, and Pd) in the mesoporous structure (Fig. 6g).

### Catalytic activity of Pd@Kryf/MCM-41

**Figure 7:**
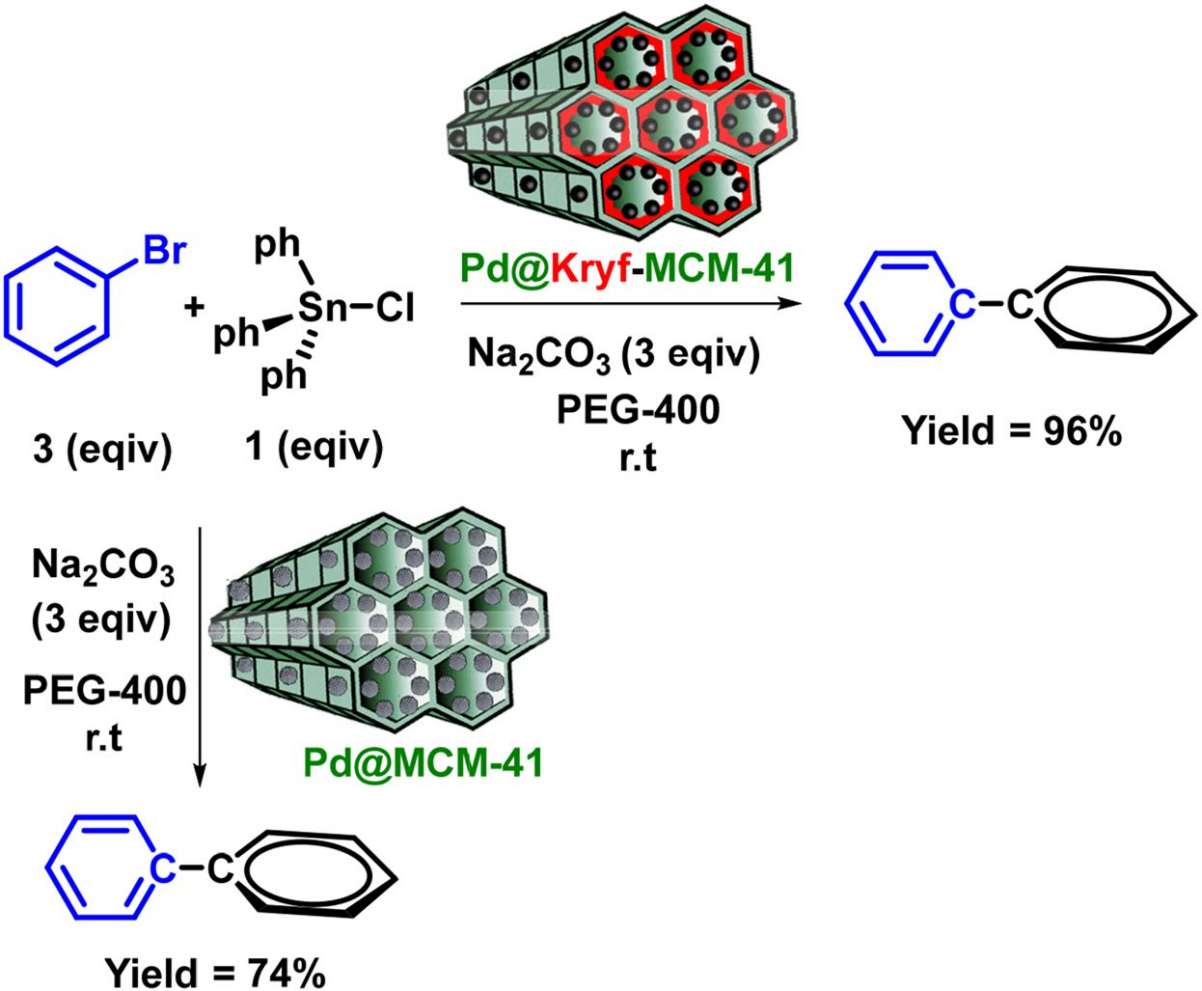
Synthesis of biaryls catalyzed by Pd@MCM-41 and Pd@Kryf/MCM-41.

The importance of the coupling reactions in pharmaceutical synthesis has led to broad interdisciplinary attention research^52,53^. On the other hand, the Stille coupling reaction due to harsh reaction conditions such as high temperature, inert atmosphere, and use of toxic solvents has caused a great challenge in the carbon–carbon coupling reactions. Therefore, after full identification of Pd@Kryf/MCM-41, employing this nanostructure for the green synthesis of biaryls was paid attention (Fig. 7).

The experiments indicated that in the absence of Pd@Kryf/MCM-41 not afford the desired product (Table 1, entry 1). When the amount of Pd@Kryf/MCM-41 increased to 0.01 g (0.36 mol% Pd) in the presence of 1 equivalent Na_2_CO_3_, the yield production was also increased up to 66% (Table 1, entry 2). Therefore, by increasing the amount of Na_2_CO_3_ to 3 equivalent, the yield was improved to 96% within 3 h, indicating a better conversion in comparison to the best catalyst systems (Table 3). However, different base was applied for the optimization such as N(Et)_3_, NaHCO_3_, CaCO_3_, NaOH, and Na_2_CO_3_. Among all the screened bases, it was observed that Na_2_CO_3_ gave a higher conversion of biaryls. After screening different solvents in optimized conditions, PEG-400 as a green solvent turned out to give the best conversion. This solvent proved to have many applications in reaction such as oxidation^54^, reduction^55,56^, addition^57^, substitution^58^, coupling^17,59^, and etc. Also, solvents nonpolar like Toluene, *n*-Hexane, and dioxane were investigated and revealed slight conversion (Table 1, entries 8–10). Besides, DMSO, DMF, EtOH, and H_2_O gave moderate conversion of biaryls (Table 1, entries 11–14). On the other hand, Pd^2+^@MCM-41 and Pd^2+^@Kryf/MCM-41 result in a moderate conversion (Table 1, entries 15 and 16). When colloidal Pd NPs immobilize in Kryf/MCM-41 with particle size 0.88 nm demonstrate that they deliver the product with more than 96% yield whitin 3 h. Ultimately, by increasing the temperature reaction from ambient condition to 120 °C, a slight increase in the yield of the reaction was observed (Table 1, entry 17), indicating the mildness and convenience of operation.

**Table 1:** Catalyst screening and reaction optimization for Stille coupling reaction^a^.

Entry	Cat	Solvent	Base	T(^o^C)	Time (h)	TOF(h^−1^)/TON^tot^	Yield (%)^b^
1	none	PEG-400	Na_2_CO_3_	25	24	–	N.R
2	Pd@Kryf/MCM-41	PEG-400	Na_2_CO_3_^c^	25	3	165/495	66
**3**	**Pd@Kryf/MCM-41**	**PEG-400**	**Na**_**2**_**CO**_**3**_	**25**	**3**	**240/720**	**96**
4	Pd@Kryf/MCM-41	PEG-400	Et_3_N	25	3	180/540	72
5	Pd@Kryf/MCM-41	PEG-400	NaHCO_3_	25	3	178/534	71
6	Pd@Kryf/MCM-41	PEG-400	CaCO_3_	25	3	172/516	69
7	Pd@Kryf/MCM-41	PEG-400	NaOH	25	3	190/570	76
8	Pd@Kryf/MCM-41	Toluene	Na_2_CO_3_	25	3	102/306	41
9	Pd@Kryf/MCM-41	*n*-hexane	Na_2_CO_3_	25	3	90/270	34
10	Pd@Kryf/MCM-41	dioxane	Na_2_CO_3_	25	3	65/195	26
11	Pd@Kryf/MCM-41	DMSO	Na_2_CO_3_	25	3	83/249	33
12	Pd@Kryf/MCM-41	DMF	Na_2_CO_3_	25	3	137/411	55
13	Pd@Kryf/MCM-41	EtOH	Na_2_CO_3_	25	3	165/495	66
14	Pd@Kryf/MCM-41	H_2_O	Na_2_CO_3_	25	3	178/534	71
15	Pd^2+^@MCM-41	PEG-400	Na_2_CO_3_	25	12	46/552	74
16	Pd^2+^@Kryf/MCM-41	PEG-400	Na_2_CO_3_	25	12	48/576	77
17	Pd@Kryf/MCM-41	PEG-400	Na_2_CO_3_	120	3	248/744	99

^a^Reaction conditions: bromobenzene (3 eqiv), ph_3_SnCl (1 eqiv), Na_2_CO_3_ (3 eqiv), PEG-400 (2 mL) in 0.36 mol% of catalyst.

^b^Isolated yield of biaryls.

^c^In presece of 1 eqiv Na_2_CO_3_.

To expand the scope of reaction substrates, a series of aryl halide derivatives were coupled with triphenyltin chloride in the presence of Pd@Kryf/MCM-41 catalyst (Table 2). The excellent conversion was obtained for all the desired biaryls without over homocoupling. Also, aryl halides with electron-donating and electron-withdrawing groups are well coupled with triphenyltin chloride, which indicates the reaction is not sensitive to the electronic effects. Besides, aryl iodides were found to be most reactive in comparison with aryl bromides and aryl chlorides, which such results were anticipated due to the high bond energy of the lighter halides^60,61^. Furthermore, the sterically hindered aryl halides (entries 3, 5, and 10 Table 2) were good tolerated under the optimal reaction conditions. These results demonstrated that this reaction was not sensitive to both steric hindrance and electron efforts, which shows a promising potential for practical application.

**Table 2:** Coupling of aryl halides with Ph_3_SnCl catalyzed by Pd@Kryf/MCM-41^a^.


Entry	Hal	Ar–H	Product	%Yield^b^	TOF(h^−1^)^c^/TON^tot^	Melting point (^o^C)	Refs.^d^
1	I	Ph-	Ph–Ph	99(97)^e^	247/742	71–72	^28,40^
2	I	*p*-Me-Ph-	*p*-Me-Ph–ph	98	245/735	45–47	^28,40^
3	I	*o*-Me-Ph-	*o*-Me-Ph–Ph	98	245/735	Oil	^28,40^
4	I	*p*-MeO-Ph	*p*-MeO-Ph–Ph	98	245/735	87–89	^28,40^
5	I	*o*-MeO-Ph	*o*-MeO-Ph–Ph	97	242/727	Oil	^28,40^
6	I	*p*-MeCO-Ph	*p*-MeCO-Ph–Ph	96	240/720	121–123	^28,40^
8	I	*m*-NO_2_-Ph	*m*-NO_2_-Ph–Ph	94	235/705	60–62	^28,40^
9	Br	Ph-	Ph–Ph	96(82)	240/720	71–73	^28,40^
10	Br	*o*-MeO-Ph	*o*-MeO-Ph–Ph	94	235/705	Oil	^28,40^
11	Br	*m*-CHO-Ph-	*m*-CHO-Ph–Ph	93	232/697	57–59	^28,40^
12	Br	*m*-NO_2_-Ph	*m*-NO_2_-Ph–Ph	90	225/675	60–62	^28,40^
13	Br	*p*-MeCO-Ph	*p*-MeCO-Ph–Ph	91	227/682	121–123	^28,40^
14	Br	1*-*Br-Naphtalene	1-Ph-Naphtalene	90	225/675	Oil	
15	Cl	Ph-	Ph–Ph	89(87)^e^	222/667	71–73	^28,40^
16	Cl	*p*-Me-Ph	*p*-Me-Ph–Ph	85	212/637	45–47	^28,40^

^a^Reaction conditions: **1** (3 mmol), **2** (1 mmol), catalyst (0.36 mol% Pd), Na_2_CO_3_ (3 mmol), PEG-400 (2 ml), r.t.

^b^Isolated yield of biaryls.

^c^TOF reaction was calculated as mmol of product formed per mmol of the catalyst at unit time.

^d^Earlier reference of the corresponding product.

^e^Pd@MCM-41 was applied.

**Figure 8:**
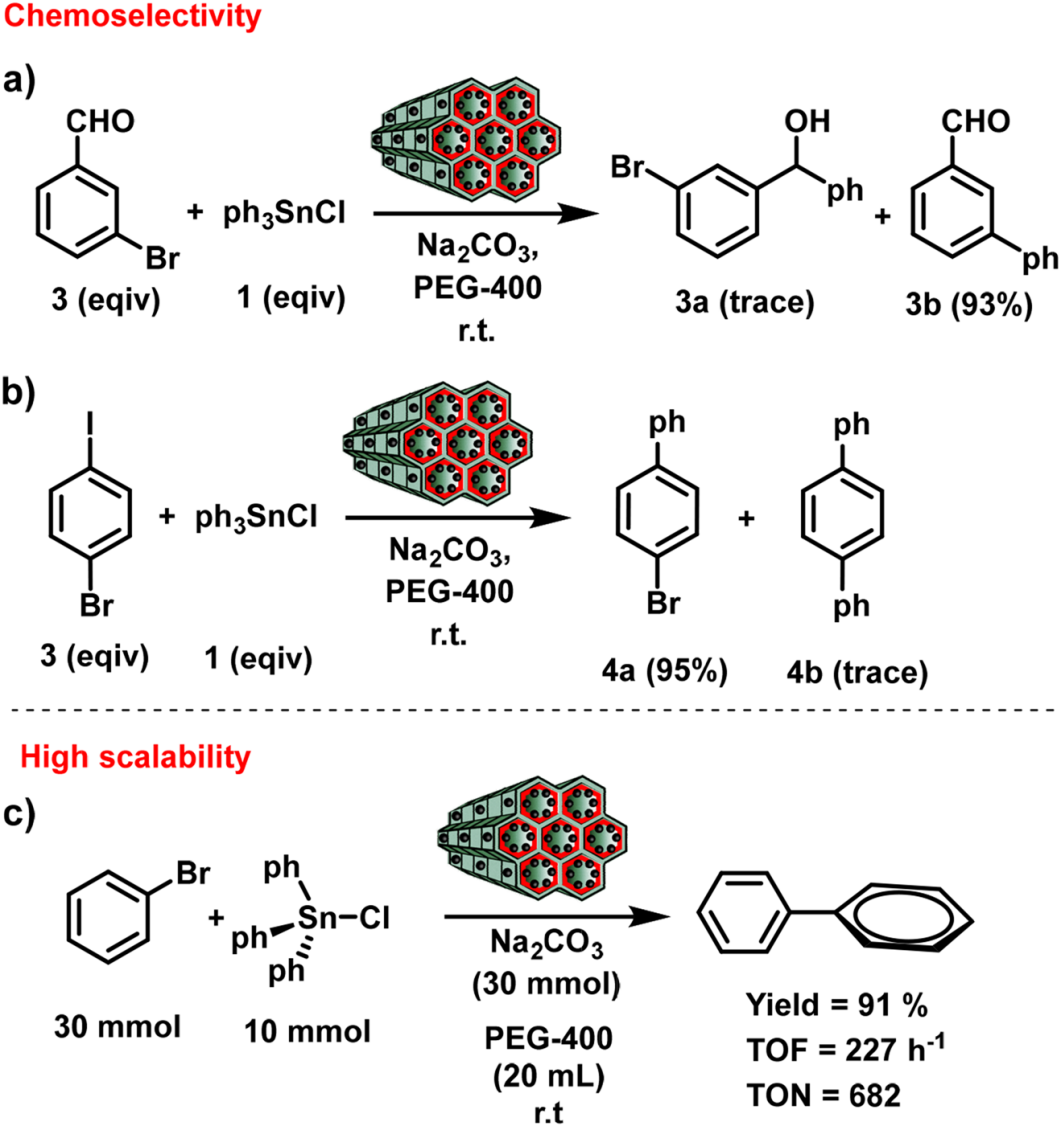
Chemoselectivity (**a**) and high scalability (**b**) of Pd@Kryf/MCM-41 in Stille coupling reaction.

In order to examine chemoselectivity, the reaction of 3-bromobenzaldehyde with triphenyltin chloride was investigated in optimize conditions (Fig. 8a). The analysis of the mixture reaction after 12 h demonstrates that biaryl product **3b** in a yield of 93% and corresponding benzophenone derivative **3a** was not observed. Moreover, chemoselectivity was further confirmed when 1-bromo-4-iodobenzene was employed as the reaction substrate (Fig. 8b). The reactions occurred exclusively with aryl iodides, but not aryl bromide and gave corresponding products **4a** with excellent yields. Furthermore, to evaluate the scalability of this catalytic system, the reaction was performed in 30 mmol scales, which conversion biphenyl in 91% (Fig. 8c). These findings indicate that the present protocol is a chemoselective, sustainable, and large-scale application.

The catalytic activity of Pd@Kryf/MCM-41 was also compared with best catalyst systems reported in the literature (Table 3). The proposed catalyst indicated an excellent catalytic activity than most of the reported works. It is evident that Pd particles in sub-nanometric scales reveal an excellent catalytic activity in terms of yield, reaction time, and catalyst load.

**Table 3:** Comparison of C–C coupling reaction with Pd@Kryf/MCM-41 and other reported systems.

Entry	Catalyst	Condition reaction	Yield (%)	Refs.
1	PdCl_2_(PPh_3_)_2_	PEG, Na_2_CO_3_,90 °C	82	^62^
2	Pd NPs	PEG, K_2_CO_3_, H_2_O, 80 °C	90	^63^
3	Pd(OAc)_2_	Dabco, Bu_4_NF, dioxane, 80 °C	93	^64^
4	Pd4 peptide	KOH, EtOH/H_2_O, 25 °C	100	^61^
5	Pd@BTU-GO	NaOAc, PEG-400, 25 °C	94	^17^
6	HMS–CPTMS–Cy–Pd	PEG, K_2_CO_3_, 100 °C	95	^65^
7	G_3_DenP-Ni	CsF, H_2_O, N_2_ atm	98	^66^
8	Pd/Fe_3_O_4_	CsF, dioxane, 100 °C	86	^67^
9	Pd _NPs_/C@Fe_3_O_4_	DMF, K_2_CO_3_, 100 °C, 25 °C	95	^68^
10	Pd@Kryf/MCM-41	Na_2_CO_3_, PEG-400, 25 °C	96	Present study
11	Pd@Kryf/MCM-41	Large-scale (30 mmol)	91	Present study

### Proposed mechanism of Stille coupling reactions

**Figure 9:**
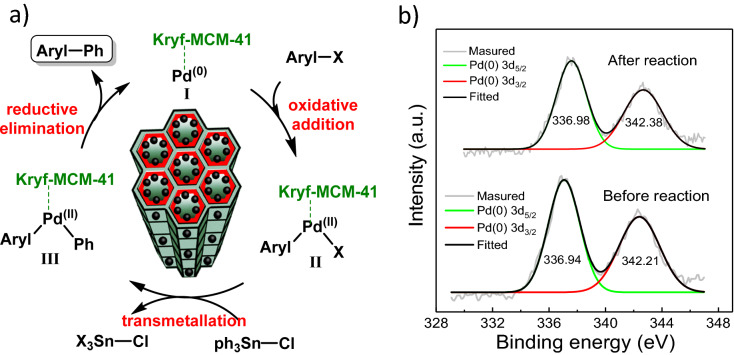
Proposed reaction mechanism for Stille reactions using Pd@Kryf/MCM-41 (**a**) and Pd 3d XPS of Pd@Kryf/MCM-41 before and after the reaction (**b**).

To further develop the mechanism of action Pd NPs in coupling reactions, a plausible mechanism for Stille coupling using Pd@Kryf/MCM-41 was presented in Fig. 9a. The oxidative addition of aryl halides to Pd^(0)^Kryf/MCM-41 is the initial step to give complex Pd and intermediate II. After that, an organotin reagent reacts with intermediate II in transmetalation to afford intermediate III. Finally, reductive elimination of intermediate III gives biaryl product and Pd^(0)^Kryf/MCM-41. In the presence of Kryf ligand, PEG-400 as a solvent, and a base (Na_2_CO_3_), probably the stable anionic complexes [Pd^0^@kryf] are formed, which can accelerate the oxidative addition of aryl halides with ph_3_SnCl^60^. Furthermore, X-ray photoelectron spectrum (XPS) analysis was used to identify the status of Pd in Pd@Kryf/MCM-41. After completing the Stille coupling reaction, the Pd 3d region exhibited two typical peaks centered at 336.98 and 342.38 eV, which can be ascribed to 3d_5/2_ and 3d_3/2_ states of Pd (0) respectively^69^ (Fig. 9b).

### Recyclability of the catalyst

**Figure 10:**
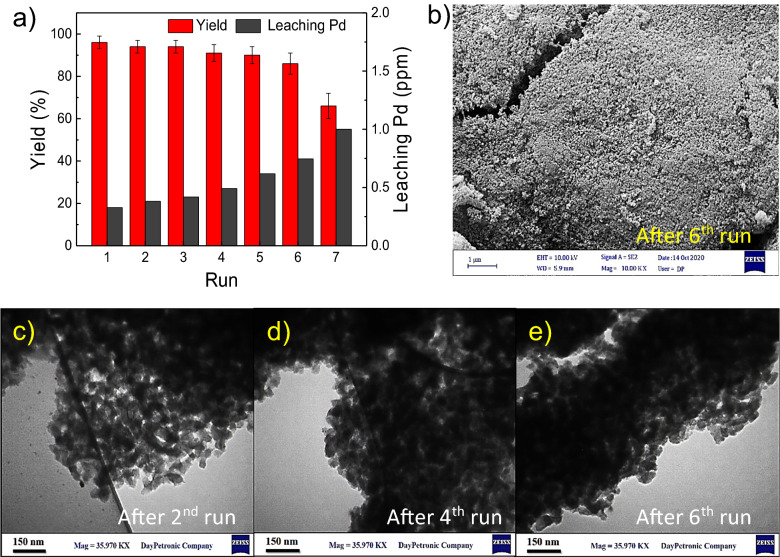
Recyclability of Pd@Kryf/MCM-41 for the synthesis of biaryls (**a**), FESEM (**b**), and TEM image of Pd@Kryf/MCM-41 (**c**–**e**).

The stability and reusability are key factors in practical application that must be considered for an effective catalytic system. To this end, the catalyst was separated by centrifuge after each use and prepared for the subsequent cycle. As shown in Fig. 10a, the activity was maintained for the first six times, suggesting that the catalyst was stable during coupling reaction. Furthermore, stable mesoporous silica material after the reuse was confirmed by FESEM (Fig. 10b) and TEM images (Fig. 10c–e). Besides, ICP-OES results indicate that after each cycle, the leaching of Pd is below 1.0 ppm (Table S2, ESI), which shows excellent physicochemical stability during Stille coupling reaction.

## Conclusions

We indicated that Kryptofix 23 could be an excellent ligand for anchoring and stabilizing sub-nanometric Pd onto the pores of MCM-41. We also expanded an efficient protocol toward the economic synthesis of biaryls under sustainable conditions. This methodology revealed that the Pd@Kryf/MCM-41 has notable advantages such as excellent stability, negligible metal leaching, large scalability, and eco-friendly nature, which could coincide with the concepts of green chemistry. We envisage that the modifying of mesoporous silica pores with crown ether compounds for the stabilization of various colloidal metals can provide a new approach for the preparation of sustainable and environmentally friendly catalysts.

## Supplementary Information

Supplementary Information.
