# Supplementary material for: A novel strategy for stabilization of sub-nanometric Pd colloids on kryptofix functionalized MCM-41: nanoengineered material for Stille coupling transformation
Source: Sci Rep. 2021 Sep 16;11:18417. doi: 10.1038/s41598-021-97914-z (PMC8446008; doi:10.1038/s41598-021-97914-z)
Supplement: Supplementary file 1 — Supplementary Information. [file 41598_2021_97914_MOESM1_ESM.docx]

**SUPPORTING INFORMATION**

**A novel strategy for stabilization of sub-nanometric Pd colloids on Kryptofix functionalized MCM-41: Nanoengineered material for Stille coupling transformation**

Hassan Alamgholiloo, ^a^ Nader Noroozi Pesyan, ^a^* Sadegh Rostamnia, ^b^*

*^a^ Department of Organic Chemistry, Faculty of Chemistry, Urmia University, 57159, Urmia, Iran.*

*^b^* *Organic and Nano Group (ONG), Department of Chemistry, Iran University of Science and Technology (IUST), PO Box 16846-13114, Tehran, Iran. Email: rostamnia@iust.ac.ir*

**Table of Contents**

**Experimental Section** S2

**Fig. S1.** TEM images of Pd@MCM-41 S3

**Table S1**. Textural properties of MCM‐41 and Pd@Kryf/MCM-41 S3

**Table S2**. ICP-OES (Pd) for Pd@Kryf/MCM-41 after each cycle of the stille reaction. S3

**Reference** S4

**Experimental Section**

**1. Chemicals and apparatus.**

Cetyltrimethylammonium bromide (CTAB, Aldrich, 99%), tetraethyl orthosilicate (TEOS, Aldrich, 99.8%), (3-chloropropyl)triethoxysilane (Merck, 95%), Kryptofix 23 (Merck, 99.5%), Pd(OAc)_2_ (Aldrich, 47.5% Pd basis), polyvinyl alcohol (Merck, 99% hydrolyzed), triphenyltin chloride (Aldrich, 95%), polyethylene glycol-400 (for synthesis), methanol (Merck, 99.5%), and ethanol (Merck, 99%) were used without further purifcation.

The morphologies of the samples were recorded on FESEM, Zeiss-SIGMA VP. TEM images were recorded on Zeiss-EM 900. The crystalline phases of the samples were analyzed by PXRD, Panalytical-Pert Pro ^'^X. FT-IR, PerkinElmer**-**Spectrum Two with an ATR probe was carried out to study functional groups. ^1^H and ^13^C NMR spectra were recorded on a Bruker AVANCE NMR spectrometers at 300 and 100 MHz, respectively.

**2. Synthesis of MCM-41 mesoporous silica**

The mesoporous MCM‐41 was synthesized through a sol–gel procedure according to our previous report [S1, S2] and elseviere [S3]. In a typical procedure, firstly, a solution containing 240 ml DI-H_2_O and 1.75 ml NaOH (2 M) was stirred at 80 °C. Next, 0.5 g (1.37 mmol) surfactant CTAB was added if the solution became homogeneous. Afterwards, 2.5 ml TEOS was slowly added dropwise into the solution which led to a white slurry. After that the obtained mixture was refluxed at the same temperature under continuous stirring for 120 min. Finally, to remove the remaining surfactant and obtain the mesoporous MCM‐41, the collected product was filtered, washed with DI-H_2_O and dried in an oven at 70 °C followed by calcination at 550 °C for 5 h with a ramp of 3 °C min^−1^.


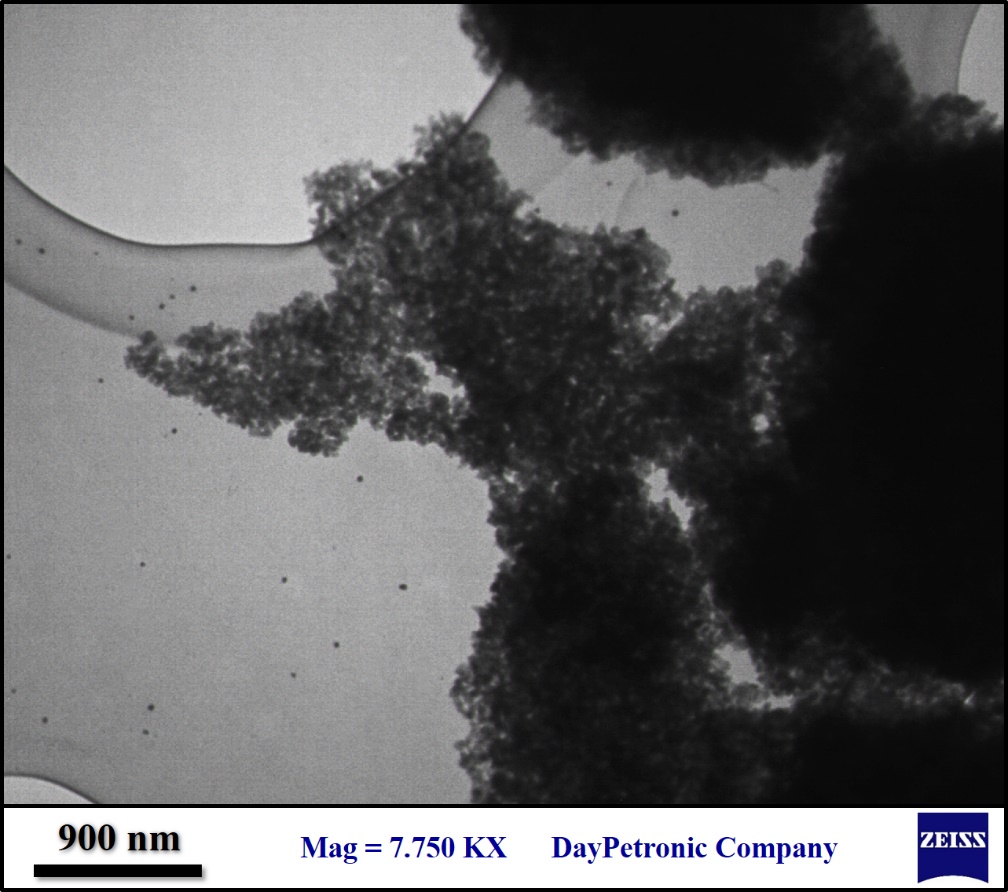


**Fig. S1.** TEM images of Pd@MCM-41

**Table S1**. Textural properties of MCM‐41 and Pd@Kryf/MCM-41.

| **Sample** | ***S*_BET_**  **(m^2^ g^-1^)** | **Pore diameter by BJH method (nm)** | **Pore volume (cm^3^ g^−1^)** | **Wall diameter (nm)** |
| --- | --- | --- | --- | --- |
| MCM-41 | 990 | 4.6 | 1.306 | 1.10 |
| Pd@Kryf/MCM-41 | 601 | 3.7 | 0.886 | 1.91 |

**Table S2**. ICP-OES (Pd) for Pd@Kryf/MCM-41 after each cycle of the Stille reaction.

| **Cycle** | **1st** | **2nd** | **3rd** | **4th** | **5th** | **6th** | **7th** |
| --- | --- | --- | --- | --- | --- | --- | --- |
| Letching Pd (ppm) | 0.319 | 0.367 | 0.421 | 0.493 | 0.611 | 0.741 | 1.019 |

**Reference**

[S1] Batmani, H., Pesyan, N. N., & Havasi, F. Ni-Biurea complex anchored onto MCM-41: as an efficient and recyclable nanocatalyst for the synthesis of 2, 3-dihydroquinazolin-4 (1H)-ones. *Microporous and Mesoporous Materials* **257**, 27-34 (2018).

[S2] Ojaghi Aghbash, K., Noroozi Pesyan, N., & Batmani, H. Cu-Kojic Acid Complex Anchored to Functionalized Silica-MCM-41: A Promising Regioselective and Reusable Nanocatalyst for Click Reaction. *ACS omega* **5**, 22099-22108 (2020).

[S3] Cai, Q., Luo, Z. S., Pang, W. Q., Fan, Y. W., Chen, X. H., & Cui, F. Z. Dilute solution routes to various controllable morphologies of MCM-41 silica with a basic medium. *Chemistry of materials* **13**, 258-263 (2001).
